# Thin Layer Drying Kinetics of By-Products from Olive Oil Processing

**DOI:** 10.3390/ijms12117885

**Published:** 2011-11-15

**Authors:** Irene Montero, Teresa Miranda, Jose Ignacio Arranz, Carmen Victoria Rojas

**Affiliations:** Department of Mechanical, Energetic, and Materials Engineering, Industrial Engineering School, University of Extremadura, Avenue Elvas s/n, 06006, Badajoz, Spain; E-Mails: tmiranda@unex.es (T.M.); jiarranz@unex.es (J.I.A.); cvrojas@unex.es (C.V.R.)

**Keywords:** olive oil residues, thin layer, drying kinetics, diffusion coefficient, activation energy

## Abstract

The thin-layer behavior of by-products from olive oil production was determined in a solar dryer in passive and active operation modes for a temperature range of 20–50 °C. The increase in the air temperature reduced the drying time of olive pomace, sludge and olive mill wastewater. Moisture ratio was analyzed to obtain effective diffusivity values, varying in the oil mill by-products from 9.136 × 10^−11^ to 1.406 × 10^−9^ m^2^/s in forced convection (*m*^a^ = 0.22 kg/s), and from 9.296 × 10^−11^ to 6.277 × 10^−10^ m^2^/s in natural convection (*m*^a^ = 0.042 kg/s). Diffusivity values at each temperature were obtained using the Fick’s diffusion model and, regardless of the convection, they increased with the air temperature. The temperature dependence on the effective diffusivity was determined by an Arrhenius type relationship. The activation energies were found to be 38.64 kJ/mol, 30.44 kJ/mol and 47.64 kJ/mol for the olive pomace, the sludge and the olive mill wastewater in active mode, respectively, and 91.35 kJ/mol, 14.04 kJ/mol and 77.15 kJ/mol in natural mode, in that order.

## 1. Introduction

The high amount of residues derived from the olive oil production, together with its temporary and highly concentrated generation, causes a serious problem in all areas from which it originated.

Spain is one of the main productive and exporting countries world-wide, producing more than 1 × 10^6^ tons/year of olive oil. According to the Agency for Olive Oil [1], Spain produced 1.11 × 10^6^, 1.24 × 10^6^ and 1.03 × 10^6^ tons of olive oil in the harvests of 2006/2007, 2007/2008 and 2008/2009, respectively. The oil extraction in the olive oil mill industry is mainly made using two systems: a three-phase or two-phase system.

In the three-phase extraction system, the residue consists of olive pomace and olive mill wastewater, whereas the latter produces an aqueous residue (moisture 90–95%, wet basis), extremely hazardous to the environment due to its high polyphenolic content [2,3]. Olive pomace constituents are pulp, olive stone, residual oil and water (moisture 50–55%, wet basis). This residue still contains residual oil (3–8% in weight) after its extraction, though for its use in small boilers it must be dried out. The most usual drying system of the olive pomace is in a rotary dryer heated up by a hot gas stream, although this operation demands a high energy consumption [2,4].

In the two-phase extraction system, the residue consists of olive sludge (moisture 60–70%, wet basis). This residue is also highly hazardous for the environment due to its high phenolic content [5]. Sometimes the sludge is three-phase re-processed in order to extract the oil contents, producing olive pomace and olive mill wastewater as residues.

The three-phase extraction system is the most commonly used in olive oil producing countries. However, from the 1991/1992 harvest, Spain mainly uses the two-phase system since it is considered to be a more ecological system as it does not produce olive mill wastewater. Nevertheless, the residue treatment problem was transferred to the extractors that had to contend with large amounts of difficult-to-process sludge.

According to olive mill wastewater and sludge characteristics, different treatments have been investigated. In the case of sludge: thermal treatments [6,7], anaerobic digestion [8], composting and bioremediation [9]; and in the case of olive mill wastewater: thermal treatments [10], aerobic treatments [11], centrifugation-ultrafiltration [12], anaerobic digestion [13], composting and bioremediation [14], fertilizer [15], photocatalysis [16], distillation [17], chemical treatments [18,19], floculation [20], ultrafiltration [21], *etc*. Currently, there is no completely efficient system for the management of these residues, and most of those existing, present some limitations that make their implementation difficult [9]. One of the treatment-assessment options is found in thermal applications, but the high energy consumption in the drying operations and various economic and technological problems limit its actual implementation.

Solar drying processes can be a very attractive technology for the treatment of olive oil mill residues in order to decrease the high energy consumption derived from the drying operations, thus decreasing the environmental impact of these residues [2,4]. In literature, there are numerous studies on the solar drying of vegetables, fruit and agricultural and agroindustrial residues [22–37]. However, there are only a few studies on solar drying at low temperature of olive oil mill residues and most of them are related to olive pomace. For instance, Akgun and Doymaz [38,39] studied the thin layer drying kinetics of olive cake (olive pomace in this work) in the drying range temperature of 50–110 °C. Gögüs and Maskan [5] researched the drying process of olive cake at 60–80 °C and Celma [2,3] studied the drying kinetics of sludge and olive husk at temperatures ranging from 20 to 140 °C.

In this work, the thin layer drying kinetics of the oil mill industry by-products (olive pomace (OP), sludge (SLG) and olive mill wastewater (OMW)) is examined. The resulting by-products, once dried out, have a final application as fuel. Therefore, the aim of this study is to determine the effective diffusivity and activation energy of the olive oil industry waste, in order to establish the feasible applicability of solar drying, as well as the influence of the operational variables on the drying time, thus allowing its actual use as a fuel.

The drying process was carried out at low temperatures, in the range of 20–50 °C, in natural and forced convection in a solar dryer designed, constructed and installed at the Industrial Engineering School in Badajoz, Extremadura (Spain).

The moisture ratio obtained (*MR*) in OP, SLG and OMW, in several tests at different temperatures and operating modes, is shown. Likewise, the effective diffusivity coefficients of moisture transfer and the activation energy for moisture diffusion have been calculated for the temperature ranges and the established operation modes.

## 2. Material and Methods

### 2.1. Material

The by-product samples of olive oil production were obtained from an oil mill by-products treatment plant located in the province of Badajoz, Spain.

The initial moisture content of OP, SLG and OMW was of (55 ± 0.50%), (70 ± 0.50%) and (90 ± 0.50%) by weight (wet basis), respectively. Six tests were carried out for each product in order to obtain a reasonable average. As can be observed, oil mill industry residues present high moisture contents, higher than agricultural residues, wood residues, sewage sludge, brown coal, peat and bituminous coal [40,41]. With the intention of comparing the drying ratios, the limit moisture established in the drying process for the three selected by-products was (20 ± 0.50%) by weight (wet basis).

Another necessary parameter for the drying kinetic study is the bulk density. Results obtained for the selected by-products before and after the drying process are shown in Table 1.

Most agricultural residues have low bulk densities. Nevertheless, if the values obtained are compared with those derived from other biomass residues, by-products from the processed olive present densities much higher than those from the grain straw (50–120 kg/m^3^), rice husk (122 kg/m^3^), industrial tomato residue (140 kg/m^3^), cork dust (285 kg/m^3^) or wood splinters (160–235 kg/m^3^) [40,41]. Related to conventional fuels [41], pomace and sludge present lower densities than those derived from the bituminous coal (800–900 kg/m^3^), and similar values to lignites (560–600 kg/m^3^), while olive mill wastewater presents a much higher density.

### 2.2. Experimental Set-Up

The solar dryer used in the experimental campaign, described previously by Celma [2] and Montero [42], consisted mainly of a flat plate collector, a drying chamber, a ventilator and a chimney. The flat collector is simple type and the drying chamber has two trays with weight sensors where the samples are placed to be dried. The moisture loss was monitored by the sensors in the trays through the drying process. The dimensions of these trays are 890 × 280 mm (*A_t_* = 0.2492 m).

The ventilator allows changing the operation mode: forced or natural convection, that is, active mode (AM) or passive mode (PM). The chimney improves the performance of the system in passive mode, thus increasing the passage speed of the drying air. The moisture ratio were obtained (*MR*) for natural and forced convection with average flows of 0.042 kg/s (passive mode) and 0.22 kg/s (active mode).

Samples were uniformly distributed on the tray as a thin layer and for each experiment their mass was kept constant, with a sample thickness of approximately 20–40 mm. The following parameters (temperature, relative humidity, air flow rate, product weight) were monitored through sensors at identical intervals in order to be tested and optimized according to Montero [42].

### 2.3. Experimental Procedure

Drying experiments were performed in Badajoz, Spain. The samples (OP, SLG and OMW) were distributed uniformly on the trays as a thin layer. Drying experiments were performed in active and passive mode with temperatures ranging from 20 °C, 30 °C and 40 °C in AM, and 40 °C and 50 °C in PM.

The tests were made for an initial amount of residue of 2000 g in each of the drying trays. Moisture loss was monitored at five minute intervals from *M_0_* (55%, 70% or 90%) to 20% of final moisture. Three replications of each experiment were carried out.

The drying data from the different samples were expressed as *MR versus* the drying time.

## 3. Results and Discussion

### 3.1. Analysis of the Drying Curves

The moisture content values obtained for the range of air temperatures of 20–30–40 °C and 40–50 °C in AM and PM, respectively, were converted into the moisture ratio, *MR*. The dimensionless moisture ratio was calculated using the simplified Equation (1) [2,43]:

(1) $$MR=M_{t}/M_{0}$$

where *M_t_* and *M_0_* are the moisture content at a given time and the initial moisture content, respectively.

**Figure 1:**
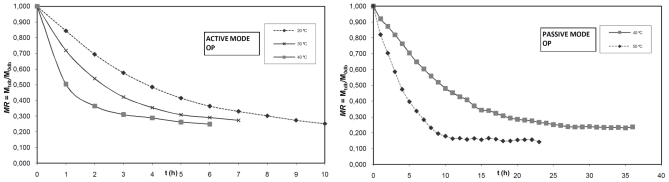

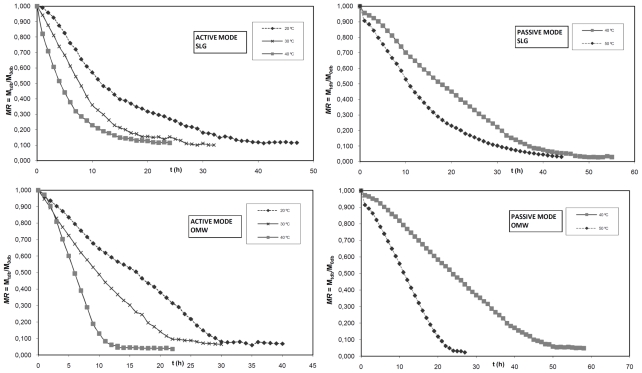
Drying curves of OP, SLG and OMW at different temperatures in AM and PM.

The evolution of the moisture ratio *versus* the drying time in AM and PM, for OP, SLG and OMW are shown in Figure 1. As seen from this figure the moisture ratio decreased continuously with the drying time, without the existence of a constant rate drying period. The total drying time was reduced along the drying air temperature increased, achieving the higher drying times in AM at 20 °C and in PM at 40 °C. These results showed that the drying air temperature and the drying mode (convective or natural) are some of the most influential factors in the drying time. Moreover, it was observed that the mass air flow exerted an influence on the drying time. Therefore, for a temperature of 40 °C, the lowest and highest drying times took place in the OP in AM and OMW in PM, respectively. These results are similar to others reported in the literature of the solar drying processes of agricultural products and olive oil residues [2,5,38,39].

### 3.2. Determination of Effective Diffusivities

The results obtained in this work pointed out that the drying time was controlled by the internal mass transfer resistance due to the existence of a decreasing rate drying period. Therefore, experimental data can be described by Fick’s diffusion equation [35]:

(2) $$MR=\frac{8}{\pi^{2}}\sum_{n=0}^{\infty }\frac{1}{{\left(2n+1\right)}^{2}}\text{exp}\left(-\frac{{\left(2n+1\right)}^{2}\pi^{2}D_{eff}t}{L^{2}}\right)$$

where *D_eff_* is the effective diffusivity (m^2^/s), L is the half-thickness of slab (m), *t* is the drying time and *n* the number of terms taken into consideration.

For long drying periods, *MR* < 0.6 [35], the Equation (2) can be simplified to the first term of the series. Considering the natural logarithm of both sides, this equation leads to the following expression:

(3) $$\text{ln}\;MR=\text{ln}\frac{8}{\pi^{2}}-\frac{\pi^{2}D_{eff}t}{L^{2}}$$

The effective diffusion coefficient is typically determined by plotting experimental data in terms of ln *MR versus* drying time, according to the equation type ln *MR = cte + Slope·t*, where:

(4) $$Slope=\frac{\pi^{2}D_{eff}}{L^{2}}$$

**Figure 2:**
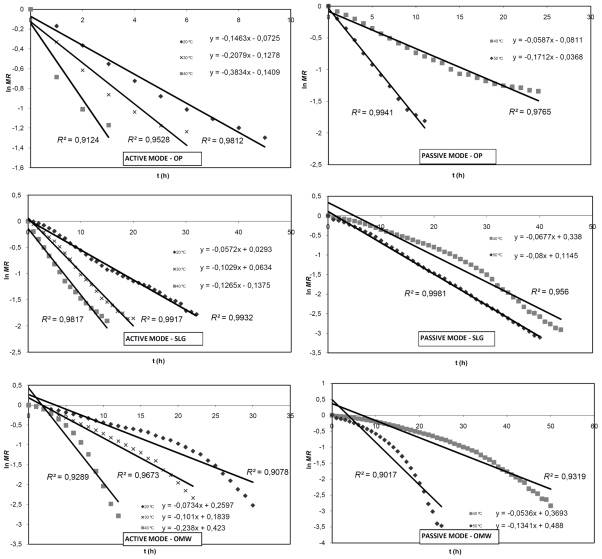
Plot of ln *MR vs.* drying time for OP, SLG and OMW in AM and PM.

Thus using the data of *MR* shown in the epigraph 3.1, Figure 2 is plotted where *Slope* values can be calculated for each temperature, residues and operation modes. For this adjustment, *R*^2^ values higher than 0.9 were obtained in all cases. Taking into account that the half-thickness of slab can be determined according to Equation (5), *D_eff_* values for different drying temperatures and operation modes are shown in Tables 2 and 3.

(5) $$L=\frac{W_{0}}{\rho \;A_{t}}$$

where *W_0_* is the initial weight of dried product (kg), ρ initial bulk density (kg/m^3^) according to Table 1, and *A_t_* the surface of product in the tray (m^2^).

As shown in Tables 2 and 3, the effective diffusivities at 20–40 °C in active mode for OP, SLG and OMW varied in the range from 9.136 × 10^−11^ to 1.406 × 10^−9^. Furthermore, at 40–50 °C and in passive mode effective diffusivities were found in the range from 9.296 × 10^−11^ to 6.277 × 10^−10^. As expected, the values of *D_eff_* increased significantly with the increasing temperature. Additionally, the values of *D_eff_* obtained in AM (Table 2) for the SLG are lower than those observed for the OMW, giving rise to AM to higher drying times in the SLG in spite of having less content of initial moisture than the OMW. The *D_eff_* values obtained are found in the usual range from 10^−11^ to 10^−9^ m^2^/s, as reported in other agricultural materials [23–27,30,31].

### 3.3. Calculation of the Activation Energy

The activation energy in a drying process, *E_a_*, is the minimum amount of energy that must be overcome for this process to occur. The value *E_a_* is closely related to the *D_eff_* coefficient and its dependence with the temperature can be expressed by an Arrhenius model [39]:

(6) $$D_{eff}=D_{0}\;\text{exp}\left(-\frac{E_{a}}{RT}\right)$$

where *D_0_* is the pre-exponential factor of the Arrhenius equation (m^2^/s), *E_a_* is the activation energy of the moisture diffusion (kJ/mol), *R* is the universal gas constant (8.314 J/(mol K)), and *T* is the air absolute temperature (K).

**Figure 3:**
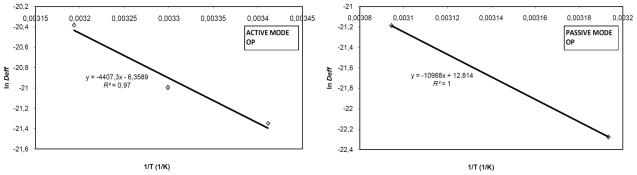

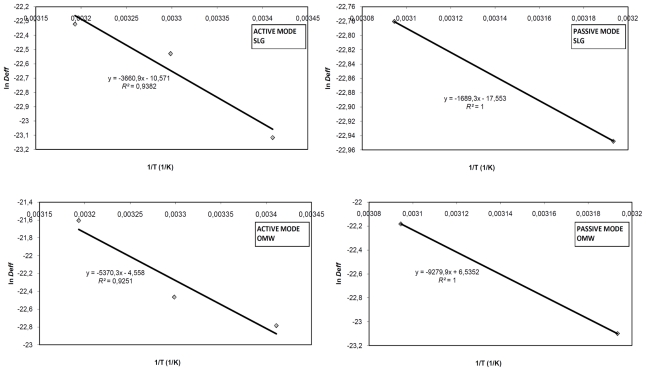
Arrhenius-type relationship between effective diffusivity and temperature for OP, SLG and OMW in AM and PM.

Equation (7) can be obtained by means of a natural logarithm. As seen from Figure 3, values of ln *D_eff_* were plotted *versus* 1/T, these data followed a linear tendency due to the Arrhenius type dependence.

(7) $$\text{ln}\;D_{eff}.=\text{ln}\;D_{0}-\frac{E_{a}}{R}\frac{1}{T}$$

The slope value of the equation above (*E_a_*/*R*) allows determination of the activation energy. Table 4 shows *E_a_* values for each by-product and operation mode obtained by means of Equation (7). In AM, the highest Ea corresponds to OMW, with 47.64 kJ/mol. On the other hand, in PM the highest value of *E_a_* corresponds to OP, with 91.35 kJ/mol. The SLG is the residue that presents the lowest value of *E_a_* in the both operation modes.

The comparison of the *E_a_* values with other agricultural and agroindustrial products reported by the literature are displayed in Table 5. As seen from this table, the activation energies of the by-products from the oil mill industry have the same order of magnitude than others found in the literature for agricultural and agroindustrial products.

## 4. Conclusions

The thin layer drying kinetics of olive oil production by-products has been investigated in this work. Olive pomace, sludge and olive mill wastewater presented initial moisture contents of (55 ± 0.5%), (70 ± 0.5%) and (90 ± 0.5%), respectively, and bulk densities of 703.2 kg/m^3^, 1065.4 kg/m^3^ and 1022.4 kg/m^3^, in that order. After the drying process in a solar dryer, a final moisture content of (20 ± 0.5%) was reached in the three by-products, and a bulk density of 559.3 kg/m^3^, 601.3 kg/m^3^ and 1035.2 kg/m^3^, respectively.

The drying time decreased as the temperature and mass flow increased. Temperature dependence on the diffusivity coefficients was described by an Arrhenius-type relationship. Effective diffusivity values of the oil mill by-products in the studied temperature ranges varied between 9.136 × 10^−11^ and 1.406 × 10^−9^ m^2^/s in forced convection, and between 9.296 × 10^−11^ and 6.277 × 10^−10^ m^2^/s in natural convection, and they increased with the air temperature.

The activation energies were found to be 38.64 kJ/mol, 30.44 kJ/mol and 47.64 kJ/mol for olive pomace, sludge and olive mill wastewater in active mode, respectively, and 91.35 kJ/mol, 14.04 kJ/mol and 77.15 kJ/mol in natural mode, in that order.

From these results, it can be concluded that the most appropriate mode of operation in the drying process is the active mode, thus decreasing the drying time of the by-products. In order to optimize the drying process, an appropriate choice of air flow is necessary to ensure an adequate drying temperature.

In general, it can be asserted that not all by-products of olive oil production are equally suitable to be dried by solar drying since, depending on the individual case, the drying times can be rather lengthy. On the other hand, the effective diffusivity values in forced convection and in the temperature range 20–40 °C are found to be higher in OP, followed by OMW and SLG, giving rise to longer drying times for the latter. Otherwise, the activation energy observed in OMW is greater than that obtained from other wastes, thus resulting in the drying process implementation being delayed.

## Figures and Tables

**Table 1:** Bulk density values of OP, SLG and OMW before and after the drying process.

	OP	SLG	OMW
Bulk density, ρ, before the drying process (kg/m^3^)	703.2	1065.4	1022.4
Bulk density, ρ, after the drying process (kg/m^3^)	559.3	601.3	1035.2

**Table 2:** Effective diffusion coefficients of OP, SLG and OMW at different temperatures in active mode.

	*D_eff_* (m^2^/s)		

Temperature (°C)	OP	SLG	OMW
20	5.364 × 10^−10^	9.136 × 10^−11^	1.273 × 10^−10^
30	7.622 × 10^−10^	1.643 × 10^−10^	1.752 × 10^−10^
40	1.406 × 10^−9^	2.020 × 10^−10^	4.128 × 10^−10^

**Table 3:** Effective diffusion coefficients of OP, SLG and OMW at different temperatures in passive mode.

	*D_eff_* (m^2^/s)		

Temperature (°C)	OP	SLG	OMW
40	2.119 × 10^−10^	1.081 × 10^−10^	9.296 × 10^−11^
50	6.277 × 10^−10^	1.278 × 10^−10^	2.326 × 10^−10^

**Table 4:** Activation energy of OP, SLG and OMW, in AM and PM.

	OP	SLG	OMW
*E_a_* (kJ/mol) in active mode	38.64	30.44	47.64
*E_a_* (kJ/mol) in passive mode	91.35	14.04	77.15

**Table 5:** Drying activation energy of various products.

Materials	*E_a_* (kJ/mol)	References
Mint	82.93	[44]
Olive cake	26.71	[38]
Sludge	15.77	[2]
Vegetable waste	19.80	[30]

## Nomenclature

*A*_t_: surface of the tray (m^2^)
AM: active mode (forced convection)
*D*_eff_: effective diffusivity (m^2^/s)
*D*_0_: pre-exponential factor (m^2^/s)
*E*_a_: activation energy (kJ/mol)
L: thickness of the slab (m)
*m*_a_: air mass flow rate (kg/s)
*M*_0_: initial moisture content (kg water/kg dry matter)
*M*_t_: moisture content at time t (kg water/kg dry matter)
*M*_0db_: initial moisture content, dry basis (kg water/kg dry matter)
*M*_tdb_: moisture content at time t, dry basis (kg water/kg dry matter)
*MR*: moisture ratio (dimensionless)
*n*: number of observations
OMW: olive mill wastewater
OP: olive pomace
PM: passive mode (natural convection)
*R*: universal gas constant (8.314 J/(mol K))
*R*^2^: coefficient of determination
SLG: sludge residue
*T*: temperature (K)
*t*: drying time (h)
*W*_0_: initial weight of dried product (kg)
ρ: bulk density (kg/m^3^)
